# Gut microbiota and microbial metabolites for osteoporosis

**DOI:** 10.1080/19490976.2024.2437247

**Published:** 2024-12-17

**Authors:** Xuan-Qi Zheng, Ding-Ben Wang, Yi-Rong Jiang, Chun-Li Song

**Affiliations:** a Department of Orthopaedics, Peking University Third Hospital, Beijing, China; b Beijing Key Laboratory of Spinal Disease Research, Beijing, China; c Engineering Research Center of Bone and Joint Precision Medicine, Beijing, China

**Keywords:** Osteoporosis, gut microbiota, bone metabolism, bile acid, SCFA

## Abstract

Osteoporosis is an age-related bone metabolic disease. As an essential endocrine organ, the skeletal system is intricately connected with extraosseous organs. The crosstalk between bones and other organs supports this view. In recent years, the link between the gut microecology and bone metabolism has become an important research topic, both in preclinical studies and in clinical trials. Many studies have shown that skeletal changes are accompanied by changes in the composition and structure of the gut microbiota (GM). At the same time, natural or artificial interventions targeting the GM can subsequently affect bone metabolism. Moreover, microbiome-related metabolites may have important effects on bone metabolism. We aim to review the relationships among the GM, microbial metabolites, and bone metabolism and to summarize the potential mechanisms involved and the theory of the gut‒bone axis. We also describe existing bottlenecks in laboratory studies, as well as existing challenges in clinical settings, and propose possible future research directions.

## GRAPHICAL ABSTRACT

**Figure:**
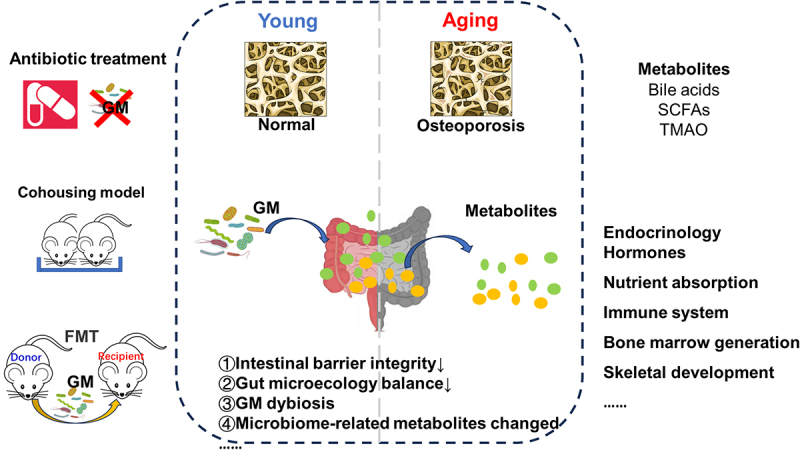

## 1. Introduction

In recent years, the aging population has led to an increase in the incidence of osteoporosis and osteoporotic fractures, resulting in a serious medical burden and a decrease in quality of life.^1,2^ Exploring the pathogenesis of osteoporosis and identifying new treatment strategies remain important endeavors for addressing this urgent scientific problem.

The intestinal microecosystem is the most abundant and complex microecosystem in the human body; indeed, the total number of microorganisms harbored in the intestine can reach 10^14^, and it may contain 150 times more genes than the human genome.^3^

The GM plays a crucial role in human metabolic health and disease. Typically, the digestion of food, the metabolism of vitamins and other nutrients, and resistance to foreign pathogens are associated with a relatively stable GM.^3^ Moreover, the GM also regulates homeostasis by affecting the human immune response^3^ and endocrine balance.^4^ However, the GM is not static; indeed, its composition, diversity and abundance of flora species can be affected by external factors (diet, medical interventions, and other exposures) or specific host diseases. Additionally, GM dysbiosis can cause endocrine and metabolic diseases, such as diabetes,^5^ intestinal disorders,^6^ and malnutrition/obesity.^7^ Relationships among the GM, the nervous system, and brain function have also been reported.^8^

Increasing attention to the concept of the GM has led to new insights that have improved our understanding of bone metabolism. Although osteopenia and osteoporosis are believed to be associated with a complex array of factors,^9^ the GM may be a candidate target for treating osteoporosis. In recent years, extensive ongoing research has indicated that changes in the GM are closely related to osteoporosis, and the potential mechanisms by which the GM affects bone metabolism include altering the relative quantities of GM-derived metabolites, regulating the host immune system, monitoring endocrine and hormone secretion, and adjusting nutrient absorption.

Among them, GM-derived metabolites originate from undigested food components and endogenous mucus secreted by intestinal epithelial cells. Following the subsequent action of the GM, several beneficial or harmful metabolites are produced, such as short-chain fatty acids (SCFAs), bile acids, surfactant derivatives, lipopolysaccharides (LPS), vitamins, and polyamines.

Notably, these microbiome-related metabolites, which have no role in the digestion of food or in nutrient absorption, participate in the regulation of cell physiology, thereby acting as a new type of signaling molecule. As the two most well-known classes of metabolites, this paper also presents an in-depth discussion on the effects of bile acids and SCFAs on bone metabolism based on the emerging literature and related studies.

This paper also introduces methods for manipulating communication and changes in the GM at the individual level under natural conditions or artificial interventions. Specifically, we describe the effects of antibiotic treatment (for the laboratory only), cohabitation (for the laboratory only), and fecal microbiota transplantation (FMT) (for both the laboratory and the clinic) on the GM and related effects on bone mass. Moreover, the effects of probiotic and prebiotic usage, exercise, medications, and diet on the GM and bone mass are also reviewed. This paper also reviews the existing challenges related to understanding how the GM and/or GM-derived metabolites affect osteoporosis, potential application strategies, and prospects for clinical application, with the aim of providing an updated view of this field and further directions for future research.

In summary, extensive evidence indicates that the GM is closely related to bone mass. At present, an increasing number of studies have focused on the specific molecular mechanisms and signaling pathways of bone metabolism that are regulated by the GM. In this paper, the relationship between the GM and bone metabolism and the potential underlying mechanisms linking the two are reviewed in combination with recent human studies and animal experiments in the field of bone metabolism. In addition, we refer to and summarize related findings from GM research in other disciplines and highlight the prospects and difficulties associated with the clinical application of diagnostic and treatment strategies based on GM regulation. We hope to bring attention to ongoing research on osteoporosis and the GM to encourage further exploration.

## 2. Concurrent changes in bone mass and the GM

### 2.1. The aging skeleton and GM

Aging has long been considered a high-risk factor and one of the main causes of osteoporosis.^10–12^ Aging is strongly linked to changes in the GM, such as impaired intestinal barrier function, bacterial translocation, decreased microbial diversity and dysbiosis.^13–19^ The promotion of 16S rDNA and metagenomic sequencing technologies has enabled researchers to establish a link between the GM and bone mass. Clinical data from cohort studies provide direct evidence supporting a role for the GM in the diagnosis and pathophysiology of osteoporosis. For example, the proportion of *Bacteroides*, a common microbe found in the microbiota, has been found to be higher in elderly individuals,^20^ although other studies have shown a decreasing trend,^21–24^ and other studies have demonstrated that the ratio of *Bacteroides* does not influence life expectancy.^24^ At the group level, the proportions of *Lactobacilli* and *Prevotella*, in addition to *Faecalibacterium prausnitzii*, are significantly lower,^23^ whereas the proportions of *Actinobacteria (Actinomycetes and Atopobium)*,^22^ *Proteobacteria* and *Bacilli* appear to be higher in elderly individuals.^24^

The results from animal experiments have shown that the relative abundances of *Prevotella, Bacteroides, Sutterella* and *Parabacteroides* are lower in aged rats, whereas those of *Bulleidia, Collinsella, Lactobacillus, Treponema* and *Allobaculum* are higher.^25^ However, sequencing data from the GM of mice vary somewhat. For example, the relative abundance of *Firmicutes* increased, and the abundances of *Bacteroidetes* and *Verrucomicrobia* decreased.^17^ In addition, experimental animals of different ages have produced varying sequencing results, even when all aged mice (e.g., 20 weeks old vs. 50 weeks old vs. 100 weeks old) present with different GM structures,^17,26^ thus reflecting age-dependent changes in the composition of the GM.

GM sequencing combined with data on bone mineral density (BMD) demonstrated that *B. stercoris*, *E. coli*, *B. uniformis*, *B. coprocola*, *B. fragilis*, *E. rectale*, and *E. eligens* correlate negatively with the T score of BMD, whereas clear positive correlations are observed between *B. vulgatus*, *B. massiliensis*, *B. caccae* and *Megamonas_unclassified* and T scores.^27^ Another bone measurement based on 18 HR-pQCT assessments suggested that four genera (*DTU089, Marvinbryantia, Blautia*, and *Akkermansia*) were negatively associated with bone parameters. However, only *Turicibacter* and *Victivallis* were consistently positively associated with bone mass.^28^ Notably, different detection methods (DXA vs. QCT), outcomes/findings (bone density vs. bone microstructure), and sites (spine vs. femur *or* tibia) have shown varying associations between specific microbial strains and bone mass. Therefore, the interpretation of these findings requires a consideration of the specific detection conditions.

### 2.2. Postmenopausal osteoporosis (PMOP)

Estrogen withdrawal is a major pathogenic mechanism of postmenopausal osteoporosis. In basic experiments, ovariectomized (OVX) female mice constitute a classical animal model used to simulate postmenopausal osteoporosis. Sequencing the GM of OVX animals revealed that the alpha diversity of the samples was similar between the sham group and OVX group or tended to increase after ovariectomy,^29^ and some studies have shown lower species richness indices in the OVX group than in the control group.^30–32^ Specifically, the abundances of *Bacteroidales*,^33^ *Bacilli*,^34^ *Gammaproteobacteria*,^35^*and Enterorhabdus*^36^ were decreased in the OVX group, and the abundances *Lachnospirales*,^33^ *Clostridia*^34^ and *Muribaculaceae* ^36^ were increased. Although different studies have reported variations in a wide variety of strains, the ratio of *Firmicutes* to *Bacteroidetes* increased after OVX.

### 2.3. Glucocorticoid-induced osteoporosis (GIOP)

GIOP is the most common type of drug-induced secondary osteoporosis. The long-term use of GCs results in rapid loss of bone mass, which is difficult to regain because of damage to the osteogenic capacity. Sequencing results have shown that, at the phylum level, the abundance of *Bacteroidetes* and *Proteobacteria* increases, whereas that of *Firmicutes* decreases.^37^ At the order level, GC treatment has been shown to decrease the relative abundance of *Verrucomicrobiales* and *Bacteriodales* and result in an increase in that of *Clostridiales*.^38^ At the family level, the abundance of *Clostridiales_vadin_BB60_group* has been reported to increase, whereas decreases in the abundances of *Bacteroidales_RF16_group*^39^ and *Defluviitaleaceae UCG-011*^40^ have been reported. One study revealed that *Parvibacter* was significantly enriched at the genus level.^41^

In addition to structural changes in the GM, some studies have suggested that energy and amino acid metabolism and lipid and phospholipid metabolism could also be affected by glucocorticoids^42^ and that intestinal barrier dysfunction is a key mediator of dysbiosis-induced bone loss.^38^

### 2.4. Other types of osteoporosis and related mechanisms

Disuse-induced osteoporosis (DIO) is another model of osteoporosis. Although changes in the external mechanical environment do not appear to directly affect the gut, simulating osteoporosis that occurs due to disuse has also been observed to significantly alter the structure of the GM. At the phylum level, the abundance of *Firmicutes* was higher in the DIO group than in the control group, and the abundance of *Bacteroidetes* was lower.^43,44^ Another study indicated that as the abundances of *Verrucomicrobia and Deferribacteres* increased, the abundances of *Candidatus, Saccharibacteria and Tenericutes* decreased.^45^ In addition, in a mouse model involving hind limb unloading, the relative abundances of *Bifidobacterium* spp. and *Akkermansia muciniphila* decreased.^46^

In conclusion, osteoporosis is accompanied by changes in the GM. Furthermore, animal experiments have shown that different osteoporosis simulation mechanisms exert varying effects on the GM. Moreover, when the same intervention (such as OVX or GCs) is used, changes in the GM at different taxonomic levels are not static. Therefore, interpreting the results of these studies requires that these factors be considered. In addition, metabolomics analyses of feces, intestinal contents, or blood can be combined with changes in the GM, providing new avenues for exploring the mechanisms of osteoporosis.

## 3. Changes in the structure of the GM to regulate bone metabolism

The evidence described previously demonstrates that changes in bone mass are accompanied by changes in the GM. In addition, structural changes in the GM have been shown to effectively regulate bone metabolism.

### 3.1. The effect of GM depletion on the skeletal system

The GM is associated with diverse and complex functions. Therefore, depleting the GM to ascertain its function and clarify its effect is an important research strategy.

Mice that underwent GM depletion were divided into germ-free (GF) mice and pseudogerm-free mice. GF mice, the gold standard for microbiota research, are mice in which no live microorganisms or parasites can be detected inside or outside the body. In contrast, compared with conventionally raised mice, pseudogerm-free mice are not truly GF mice but have nearly no bacteria in their guts. Pseudogerm-free mice are generally obtained after special treatment of SPF mice^47–50^ with an antibiotic cocktail (ABX), and these mice are also known as antibiotic-treated (ABT) mice. No standard formula is available for this cocktail, and antibiotics, including ampicillin (1 g/l), metronidazole (1 g/l), neomycin trisulfate (1 g/l), and vancomycin (0.5 g/l), are commonly used in combination.^51^

Although GF mice do not exhibit additional immune responses associated with antibiotic use, the disadvantages of GF mice are their slow breeding time and expense. As a result, ABX mice are more widely used in research as effective alternative models for GF mice.

Compared with conventionally raised mice, GF mice congenitally exhibit special characteristics in terms of growth,^52–54^ immunity,^55,56^ metabolism^57–59^ and nutrient absorption.^60,61^ After depletion of the GM, ABX mice also exhibit changes in some physiological functions of the body and subsequently exhibit different responses to pathogenic factors^62–64^ and drugs.^65–67^

In terms of bone mass, GF mice exhibited reduced bone resorption compared to conventionally raised mice. Decreases in the number of osteoclasts on the bone surface of GF mice, the frequencies of CD4^+^ T cells and CD11b^+^/GR1^+^ osteoclast precursor cells in the bone marrow, and the expression of inflammatory cytokines have been observed.^68^ Another study confirmed that GF mice have an increased trabecular bone microarchitecture and increased tissue strength but reduced whole-bone strength.^69^ One study revealed that GF mice had a lower bone mass than conventionally raised mice, which the authors posited was related to the IGF-1-mediated growth axis, indicating that the GM maintains tissue growth.^70^ The genetic background was also responsible for the difference in bone mass between GF and conventional mice. Interestingly, the GM is also implicated in estrogen deficiency-induced bone loss. In GF mice, researchers have shown that sex steroid deficiency does not cause an increase in osteoclasts and does not cause the loss of trabecular bone.^71^ Our team also verified this finding in an OVX model in pseudogerm-free mice.^72^ ABX-treated mice also presented reductions in tissue mechanical properties and total bone strength, with only minor changes in bone geometry and density.^73^ However, the bone mass findings in the ABX mice varied. Indeed, some studies have reported a decrease in bone mass in ABX mice compared with controls.^38^ One possible explanation for these contradictory results is that studies were conducted in mice with different genetic backgrounds.

The sexually dimorphic effect of GM depletion on bone mass is worth noting. An increased microstructure of the trabecular bone has been observed in GF mice, but this effect is more pronounced in males than in females.^69^ Additionally, structural parameters of trabecular and cortical bone have been reported to decrease in male C57BL/6J mice after neonatal ABX administration, whereas the effects of ABX on female mice are more moderate.^74^ Both studies reported that early exposure to antibiotics in neonates (P7–P23)^47^ or early childhood (3 weeks old)^47^ influenced bone mass loss in adulthood.

ABX mice are widely used to explore the effects of the GM on osteoporosis. GM depletion significantly reduces bone loss after ovariectomy,^72,75,76^ possibly due to increases in the *Firmicutes:Bacteroidetes* ratio after OVX, a phenomenon that can be eliminated with ABT. In addition, decreases in serum LPS and inflammatory factor levels and improvements in metabolism are potential mechanisms. However, these findings are clearly insufficient, as the causes underlying changes in the GM after the host has undergone ovariectomy and the identification of responsible strains and systemic changes after eliminating the GM are all questions worth answering.

In addition to changes in bone mass, depletion of the GM affects other aspects of the locomotor system. Compared with the sham group of mice, both ABX mice and GF mice presented decreased voluntary wheel running behavior. Moreover, the age of ABX mice (6 weeks) did not affect their ability to adapt to endurance training, but GF mice showed decreased responsiveness to endurance exercise training.^77^ This property may be due to developmental defects associated with a lack of microbes at birth. In addition, both ABX mice^75,78^ and GF mice^79^ presented reduced inflammation and symptoms of knee osteoarthritis.

### 3.2. Probiotics and prebiotics

According to the latest International Scientific Association for Probiotics and Prebiotics (ISAPP) consensus statement, probiotics are defined as live microorganisms that, when administered in adequate amounts, confer a health benefit on the host.^80^ Prebiotics are substrates that are selectively utilized by host microorganisms and confer health benefits.^81^

Probiotics can accumulate in the gut and play a beneficial role by inhibiting the colonization of pathogenic bacteria and by actively regulating the balance of the bacterial composition. Prebiotics can selectively promote the metabolism and proliferation of beneficial bacteria in the gut and indirectly maintain intestinal homeostasis. Prebiotics include carbohydrates such as polysaccharides and fructose, as well as noncarbohydrate substances such as polyphenols and fatty acids.

Both probiotic and prebiotic supplementation can alter the structure and composition of the GM and improve bone mass, possibly by promoting the proliferation and metabolism of beneficial bacteria in the intestine, by inhibiting the colonization of intestinal pathogens, by actively regulating the balance of the bacterial composition, and by maintaining intestinal homeostasis. Therefore, with prebiotic and probiotic usage, the integrity of the intestinal mucosal barrier is maintained, host immune function is regulated, and intestinal metabolism and nutrient absorption are regulated.

*Akkermansia* is a widely known probiotic that plays a significant role in anti-inflammatory, antiobesity and other processes.^82,83^ It is also considered of potential value in the prevention and treatment of osteoporosis. Compared with young mice, old mice presented the most significant reduction in the abundance of *Akkermansia*.^26^

Although several animal studies of probiotics and prebiotics for bone health have been conducted, clinical trials examining individual probiotics/prebiotics are rare. A randomized controlled trial (RCT) of 90 people revealed that *Lactobacillus reuteri ATCCPTA 6475 (L. reuteri 6475)* reduced bone loss in total tibial volume BMD (vBMD) after 12 months of intake, but this difference was not statistically significant.^84^ The team then conducted a study of 20 female subjects, and when the greatest changes (good responders) and the lowest changes (poor responders) in total tibial volume BMD were compared, the results revealed that *L. reuteri 6475* supplementation improved bone density by increasing the gene richness, composition and function of the GM and SCFA production.^85^ Further research by the team revealed that amino acid metabolism, peptide metabolism and lipid metabolism were altered after *L. reuteri 6475* supplementation, among which the level of butyrylcarnitine in particular strongly increased, indicating that the bone-protective effect of *L. reuteri 6475* was mediated by butyrate signaling.^86^

In summary, although probiotics and prebiotics have positive protective effects on bone, the underlying mechanism is multifactorial and complex, and clinical studies with a high level of evidence are lacking. Given the current widespread use of probiotics and prebiotics as health products and their great potential to treat osteoporosis, more clinical trials and laboratory studies exploring the underlying mechanism are urgently needed.

### 3.3. Cohousing mouse model

The GM varies greatly among host individuals. Variations in the strain, feeding facility, breeding methods, and microflora in the body may be the reasons for the heterogeneity observed across studies. Therefore, GM-related studies in particular must ensure good uniformity across samples/participants at the group level. Moreover, promoting gut microecology homogenization among experimental animals is another experimental approach that can be taken.

Cage-sharing experiments are simple and universal methods for homogenizing microbial communities. The cohousing mouse model utilizes the practice of coprophagy and the habit of cleaning the body by ingesting feces to achieve horizontal transmission of intestinal microbes between caged animals.

One study reported that the migration of microbial communities changed dynamically with time after cohousing. After 7 days of cohousing, microbial communities (at the level of bacterial OTUs) tended to be roughly homogenized among different host populations.^87^ This study provides direct evidence and a theoretical basis for the role of cohousing in gut microbial exchange. Another study revealed that even caged mice in which coprophagy was prevented had lower alpha diversity and metabolic, neurochemical, and cognitive impairments than did mice that were engaged in coprophagy freely.^88^

Few studies on orthopedic diseases have examined the effects of cohabitation. Cohousing with healthy mice attenuated osteonecrosis by altering the gut microbiome in a mouse model of glucocorticoid-induced osteonecrosis of the femoral head.^89^ In another study, GF rats cohabitated with conventional rats. After 10 days, the gut microbiota of GF rats significantly changed, and the accumulation of bone in the bone cortex and trabeculae accelerated.^90^ In addition, elevated serum levels of 25-hydroxyvitamin D and alkaline phosphatase indicated active bone formation. In contrast, colonizing the microbiota of conventionally bred mice into GF mice, rather than cohabitating them, resulted in recipient mice with a reduced trabecular bone mass at 1 month postcolonization compared with that of GF controls.^91^

However, cohousing, as a rejuvenation procedure, affects the structure and function of gut microbes, reduces age-related intestinal inflammation, improves intestinal barrier integrity, and reverses age-related phenotypes.^26^ Although young mice are rejuvenated, young mice may also suffer from premature aging via mechanisms that are related to the gut microbiota.^92^

The role of cohousing extends beyond gut microbiota exchange. The living environment of the same cage can be understood as a small society. Positive social networks have been linked to adequate maintenance of health and slower aging. Prematurely aged mice (PAM) cohoused with special nonprematurely aged mice (NPAM) presented improved immune function in the spleen and thymus, improved redox status in the liver and heart and ultimately an extended lifespan.^93^

To delve deeper into reality, short social interactions between the PAM and NPAM may underly the observed improvements in health.^94^ Furthermore, physical contact appears to be crucial to experiencing the positive effects of social interaction.^95^ In addition, studies have suggested that social interaction rescues memory deficits in animal models of Alzheimer’s disease by increasing brain-derived neurotrophic factor (BDNF)-dependent hippocampal neurogenesis.^96^

In real human society, the idea that social interaction promotes healthy aging is well established. Cohabitation is also used as a method of intervention and as a research model to study healthy aging,^97–99^ but it is fundamentally different from the cohousing mouse model because of the coprophagic nature of mice and because this type of gut microbiota exchange is impossible to reproduce in humans. Another important point is that although the effect of the gut microbiota is limited in real human society, other stimuli between individuals, such as sound and light/scenes, need to be considered.

In conclusion, cohousing is a simple and effective strategy for achieving homogenization that is similar to natural gut microbial exchange. Currently, cohousing is rarely used in orthopedic research, and even in cohousing mouse models that involve cohousing old and young animals together, researchers have seldomly examined changes in bone mass. Future studies may explore the effects and mechanism of cohousing on bone and use a cohousing mouse model as a means to improve bone quality.

### 3.4. Fecal microbiota transplantation (FMT)

FMT involves the transplantation of functional intestinal flora from the stool of a specific healthy donor into the intestinal tract of a patient with the aim of reshaping the dysbiotic GM of the recipient to treat intestinal^100–102^ and extraintestinal diseases.^103–106^

#### 3.4.1. Effect of FMT on bone mass

Four weeks after the transplantation of intestinal bacteria from normally raised mice to GF mice, the bone mass of GF mice post-transplantation (Trans-GF mice) was lower than that of GF mice.^72^ This finding agrees with previously mentioned observations showing that the bone mass of GF mice is greater than that of normally fed mice.^68^ Another study revealed that bone mass in GF mice decreased one month after transplantation but increased 8 months after transplantation and gradually returned to the level of normal mice.^91^ The decrease in bone mass in GF mice one month after FMT may be due to the activation of the immune system.^68,107,108^ The return of bone mass to the normal level after FMT may be due to changes in the GM caused by prolonged feeding (8 months), which indirectly affects bone mass. Overall, this innovative study has several implications. First, the results indicate that the effect of gut microbiota transplantation on bone mass is time sensitive and that the short- and long-term effects are different; therefore, the variable of time must be considered when evaluating FMT effectiveness. Second, FMT can be used to regulate bone mass. Evidence from animal experiments shows that transplantation of the GM from young mice aged 3 months into 18-month-old older mice increases bone mass by improving the gut microbiome composition and gut barrier function.^109^ Transplanting intestinal microbes from healthy mice prevents OVX-induced bone loss by correcting intestinal microbial imbalances, optimizing intestinal permeability, and inhibiting the release of osteogenic cytokines (TNF-α and IL-1β).^110^ Another study conducted in rats also showed that FMT from normal rats improved osteoporotic bone loss in OVX rats. The underlying mechanism involves the activation of the brain‒gut‒bone axis by the neuroendocrine signal neuropeptide Y (NPY), which regulates the microbial diversity of OVX rats and alters the community composition of the gut microbiota, thus affecting the entry of gut microbiota-related metabolites (such as LPS) into the blood circulation.^33^

Animal studies have also been conducted using the healthy GM from humans. For example, transfer of the GM from children, rather than from older individuals, into OVX mice prevented OVX-induced bone loss.^111^ It is worth noting that this research also suggests that the underlying cause is the restoring the abundance of *Akkermansia muciniphila* in the GM of OVX mice. Further studies also confirmed that extracellular vesicles released by *Akkermansia muciniphila* may directly promote bone formation and inhibit osteoclastic absorption, and alleviates the bone microstructure degeneration and decrease in bone mass caused by estrogen deficiency. This study revealed a novel “gut-bone axis” bone metabolism regulation mode mediated by functional extracellular vesicles of GM, laying a foundation for the future use of probiotics or their functional extracellular vesicles to prevent and treat osteoporosis and its complications.

In addition to treatment effects, FMT has also been used to study the adverse effects of a gut microbiota imbalance. Transplanting gut microbes from old mice into young mice for 12 weeks resulted in decreased bone mass in recipient mice, possibly due to an impaired intestinal barrier caused by the dysregulation of the gut microbiota, leading to osteoporosis.^112^ However, transplantation from older donors did not significantly reduce bone mass in younger recipient mice.^113^ In addition, recipient mice that received gut microbe transplants from old mice may have advantages in terms of hippocampal neurogenesis, intestinal surface area expansion, and butyrate metabolism compared with recipient mice transplanted with gut microbes from young mice.^114^ In another trial, when gut microbes from GIOP mice were transplanted into normal mice for one week, the recipient mice experienced a reduction in bone mass, suggesting that the effects of glucocorticoids on bone mass could be transferred via FMT methods.^38^

#### 3.4.2. Anti-aging effect of FMT

Although few animal experiments or clinical trials have examined FMT for the treatment of osteoporosis, FMT is another research hotspot in the antiaging field. Various studies have shown that FMT can reverse brain, skin, intestinal and systemic inflammation (Table 1). Osteoporosis is a manifestation of aging in the skeletal system. As the antiaging process progresses, bone mass may also improve. Unfortunately, the above studies did not mention changes in the skeletal system. Therefore, the application of FMT in the treatment of osteoporosis may be worth exploring in the future.

**Table 1.** FMT studies in antiaging fields.

Number	Disease/model	Donors	Recipients	Aims/results	Mechanism	Methods and frequency	ABX before FMT?	Ref
1	Stroke	C57BL/6 mice (8–12 weeks)	C57BL/6 mice (18–20 months)	Aged recipient mice treated with a young microbiota had decreased mortality after MCAO.	In the context of stroke, bidirectional communication of the microbiota – gut – brain axis exists. The negative effects of an aging microbiota may involve an enhanced inflammatory response after stroke.	Gavage daily for 5 days.	Gavage of antibiotics consisting of 500 mg of streptomycin HCl/ml of sterile water for 2 consecutive days.	115
2		C57BL/6 mice (18–20 months)	C57BL/6 mice (8–12 weeks)	Young mice treated with an aging microbiota had increased mortality after MCAO.				
3	Neurogenesis	C57BL/6 mice (24 months)	Young GF mice (5–6 weeks)	However, recipient mice transplanted with gut microbes from old mice may have advantages in hippocampal neurogenesis, intestinal surface area expansion, and butyrate metabolism compared with recipient mice transplanted with gut microbes from young mice.	Transplantation of the gut microbiota in older mice increases the abundance of butyrate-producing bacteria and the availability of butyrate in younger recipient mice, thus promoting a butyrateFGF21-driven metabolic profile.	Gavaged. Repeated on day 3 after the initial inoculation.	GF mice	114
4		C57BL/6 mice (5–6 weeks)	Young GF mice (5–6 weeks)					
5	Gut immune system	C57BL/6 and BALB/c mice (3 months)	C57BL/6 and BALB/c mice (22 months)	FMT promotes the gut germinal centers of intestine regardless of age directionality.	The poor germinal center response in older animals can be improved by providing FMT as an appropriate stimulus.	Oral gavage on days 1, 6, 9, 13, 16, and 20.	N.A.	116
6		C57BL/6 and BALB/c mice (21 months)	C57BL/6 and BALB/c mice (3 months)					
7	Healthspan and lifespan extension	Wild-type mice (4 months)	Premature aging mice (*LmnaG609G/G609G*, 8–10 weeks)	After receiving FMT, the survival rate of premature aging mice was improved.	After FMT treatment, secondary bile acids and other metabolites (arabinose, ribose, and inosine) may help extend the healthy lifespan and longevity in prematurely aged mice.	Gavage twice a week for two weeks, and then once a week until death.	antibiotic cocktail with 1 g/L ampicillin, 0.5 g/L neomycin, 0.5 g/L vancomycin and 1 g/L metronidazole for three consecutive days.	117
8		Premature aging mice (*LmnaG609G/G609G*, 4 months)	Premature aging mice (*LmnaG609G/G609G*, 8–10 weeks)	After receiving FMT, the survival rate of premature aging mice was reduced.				
9	Cognitive decline	Rats (20–24 months)	Rats (3 months)	FMT of the aging microbiota in older rats impairs cognitive behavior in younger rats.	FMT of the aged microbiota increases inflammatory cytokine levels and oxidative stress levels in young rats.	Gavage. The rats underwent FMT once a day for three days, and then twice a week for two months.	Antibiotic cocktail with ampicillin (180 mg/kg/d), vancomycin (72 mg/kg/d), metronidazole (90 mg/kg/d) and imipenem (90 mg/kg/d), twice daily (12 h interval) for three consecutive days.	25
10	Brain lipid metabolism	C57Bl/6JRj mice (4 weeks)	Germ-free C3H/HeN mice (8 weeks)	The intestinal flora influences the contents of cholesterol and phospholipid in the cortex.	Changes in the gut microbiota associated with aging affect lipid metabolism in the brain and liver.	Gavage.	GF mouse	118
11		C57Bl/6JRj mice (24 months)	Germ-free C3H/HeN mice (8 weeks)					
12	Cognitive functions	C57BL/6 mice (24 months)	C57BL/6 mice (3 months)	FMT from older donors results in impaired spatial learning and memory in young adult recipients.	Significant decreases in the abundance of bacteria associated with SCFA production and central nervous system disorders was observed. The microglia of the hippocampal pili acquire an aging-like phenotype.	Oral gavage was performed six times on days 24–28 and 35 from the beginning of the antibiotic regime.	The mice were given antifungal treatment with amphotericin B 1 mg/kg daily on days 1–3, and force-fed metronidazole 100 mg/kg daily on days 4–17, while the antibiotic mixture (ampicillin 1 g/L, vancomycin 0.5 g/L and neomycin 1 g/L) was added to drinking water. On the 18th to 24th day, ampicillin 1 g/L, vancomycin 0.5 g/L, neomycin 1 g/L, metronidazole 100 mg/kg, amphotericin B 1 mg/kg were orally fed daily.	119
13	Stroke recovery	Nonstroke young mice (2–3 months)	Aged mice with MCAO (18–20 months)	Poor stroke recovery in older mice can be reversed with poststroke bacterial therapy.	Young fecal transplants contain higher levels of SCFAs and related bacterial strains, and FMT increases intestinal, brain and plasma SCFA concentrations in elderly recipients, alleviating neurological deficits and inflammation after stroke.	Oral gavage at days 3 and 4.	Gavage with streptomycin for two consecutive days.	120
14		Nonstroke aged mice (18–20 months)	Aged mice with MCAO (18–20 months)					
15	Osteoporosis	Rats (3 months)	Rats (18 months)	FMT reduces bone loss in older rats.	FMT improves the intestinal structure and upregulates the expression of closure protein, claudin and ZO-1 tight junction proteins.	Oral gavage three times per week for 12 weeks or 24 weeks.	N.A.	109
16	Lacrimal gland circadian dysfunction	C57BL/6 mice (5–6 weeks)	C57BL/6 mice (20 months)	Chronic inflammation, lipid deposition, and abnormal neural responses in aging lacrimal glands are significantly reduced.	FMT therapy can restore associated pathways and characteristic functional changes associated with aging and disease.	Oral gavage three times a week for 4 consecutive weeks.	N.A.	121
17	Cognitive behavior	C57BL/6 mice (10–12 weeks)	C57BL/6 mice (19–20 months)	The young donor-derived microbiota mitigates selective age-related impairments in cognitive behavior in older recipients.	Transplanting the microbiota from young donors reverses age-related changes in peripheral and brain immunity, as well as the hippocampal metabolome and transcriptome of aging recipient mice.	Oral gavage. FMT occurred once per day for the first three days to encourage microbiota engraftment and then twice per week thereafter.	N.A.	122
18	Intestinal barrier dysfunction	C57BL/6J mice (3 months)	C57BL/6J mice (17 months)	Transplanting fecal microbiota into older mice does not prevent aging-related gut barrier dysfunction in the small intestine.	Damage to the gut barrier level may not be primarily caused by changes in the composition of the gut microbiota. The possible reasons are the change in intestinal NO homeostasis, especially the increase in arginase activity and the decrease in NO bioavailability in intestinal mucosa.	Oral gavage three times weekly for the following six weeks.	Antibiotic cocktail with polymyxin B (92 mg/kg BW) and neomycin (216 mg/kg BW) in drinking water for three days.	123
19	Skin aging	C57BL/6 mice (5 weeks)	C57BL/6 mice (12 months)	FMT derived from gut microbes in young mice thickens the stratum corneum, increases the thickness and grip strength of muscle fibers, and enhances the skin’s ability to retain water.	Dbn1 is induced by a young microbiome and regulates skin hydration.	Oral gavage twice a week for a total of 8 weeks.	Drinking water containing antibiotics (1 g/L ampicillin, 0.5 g/L vancomycin, 1 g/L metronidazole, 1 g/L neomycin, and 2.5 mg/L amphotericin B) for 7 days.	124
20	Locomotor and exploratory abilities	SAMP8 mice (2–3 months)	SAMP8 mice (7 months)	Young gut microbes delay the decline in motor ability and the number of objects explored by older mice.	The increased beta diversity of gut microbes, as well as the relative abundance of *Akkermansia*, may also increase the body’s resilience to metabolic aging	Gavage once a week.	No ABX.	125
		SAMP8 mice (10–11 months)	SAMP8 mice (7 months)					
21	Systemic inflammation	C57BL/6J mice (3 months, 18 months, and 24 months)	C57BL/6J mice (3 months)	Age-related changes in the gut microbiome, disruption of the integrity of the gut barrier, and systemic and tissue inflammation affecting the retina and brain can be reversed by replacing it with a younger donor microbiome.	Heterochronous FMT reverses age-related breakdown of epithelial barrier integrity and systemic inflammation, along with altered lipid and vitamin metabolism.	Twice, 72 h apart by oral gavage.	Antibiotic cocktail with 5 mg/mL vancomycin and 10 mg/mL metronidazole for 3 days; and drinking water containing 1 g/L ampicillin and 0.5 g/L neomycin.	126
22		C57BL/6J mice (3 months, 18 months, and 24 months)	C57BL/6J mice (18 months)					
23		C57BL/6J mice (3 months)	C57BL/6J mice (24 months)					
24	Ovarian function	C57BL/6 mice (5 weeks)	C57BL/6 mice (42 weeks)	Fecal microbiota transplants from young donor mice improve ovarian function in older mice by reducing follicular atresia and apoptosis, and increasing ovarian cell proliferation.	The gut microbiota composition of the FMT-treated mice shows a “young-like phenotype” and an increase in symbiotic bacteria. In addition, the FMT-treated mice show an increase in the level of the anti-inflammatory cytokine IL-4 and a decrease in the level of the pro-inflammatory cytokine IFN-γ.	Oral gavage three times one week for eight weeks.	Drinking water (1 g/L ampicillin, 0.5 g/L vancomycin, 0.1 g/L gentamicin, 0.01 g/L erythromycin, and 0.5 g/L neomycin) for two weeks.	127
25	Osteoporosis	Rats (18 months)	Rats (3 months)	Bone mass decreases and bone turnover accelerates in recipient rats (3 months).	The intestinal structure is damaged in FMT rats, and the expression of occludin, claudin, and ZO-1 proteins decreases.	Oral gavage three times a week for 12/24 weeks.	N.A.	112
26	Antioxidant defenses	C57BL/6 mice (5 weeks)	GF C57BL/6 mice (4 weeks)	Transplanting the aged transgene into young adolescent GF mice has no effect on lipid peroxidation, but enhances free radical scavenging in the heart and liver.	Changes in antioxidant defenses associated with aging may be associated with four gut microbial genera (*Akkermansia, Dubosiella, Alistipes, and Rikenellaceae_ RC9_gut_group*).	Oral gavage on days 1, 4, 7, 14, and 21.	GF mice	128
27		C57BL/6 (20 months)	C57BL/6-GF mice (4 weeks)					
28	Cancer	C57BL/6 mice (6 weeks)	A/J mice (8 weeks)	Compared with recipients of the microbiota from younger donors, recipients of the microbiota from older donors have a higher incidence of colonic tumors, higher colonic proliferation and higher levels of inflammatory cytokines.	The expression of mitochondria-related genes is significantly increased in the recipient of the microbiota from aged donors.	Three FMTs were conducted in 1 week and every 4 weeks thereafter for 5 months.	Antibiotic cocktail with neomycin (1 g/L), vancomycin (0.5 g/L), metronidazole (0.5 mg/L) and ciprofloxacin (0.125 g/L) in drinking water for 7 days.	129
29		C57BL/6 mice (72 weeks)	A/J mice (8 weeks)					
30	Osteoporosis and sarcopenia	C57BL/6J mice (21 months)	C57BL/6J mice (5 months)	Compared with the gut microbiota from young adult donors, the gut microbiota from older mice reduces the percentage of lean body mass in recipient mice, but does not reduce bone mass.	The presence of *Bacillus ovalis* in the cecum/stool is a marker of a high relative lean body mass in mice and humans.	Oral gavage at 5, 11, or 17 weeks of age.	GF mice	113
31	Spermatogenic dysfunction	C57BL/6J mice (18–20 months)	C57BL/6J mice (6 weeks)	Heterochronic FMT mitigates the decline of spermatogenesis in old mice.	Intestinal microbiome transplantation and the intestinal microbial-derived metabolite 3-HPAA promote spermatogenesis in older mice through the inhibition of GPX4-induced ferroptosis.	FMT treatment at 1-day intervals for 6 weeks.	Antibiotic cocktail with 0.5 g/L vancomycin, 1 g/L ampicillin, 1 g/L kanamycin, and 1 g/L metronidazole for 3 days.	130
32	Hematopoietic stem cell aging	CD45.2 or CD45.1 C57BL/6 mice (7–8 weeks)	CD45.2 or CD45.1 C57BL/6 mice (20–24 months)	FMT from young mice improves the defective phenotype of elderly HSCS, restores the recombination ability of elderly HSCS, reduces inflammation, activates the FoxO pathway, and promotes the lymphatic differentiation of elderly LT-HSCs.	FMT from young mice protects the integrity of the gut barrier in older mice. Lachnospiraceae, tryptophan and indole-3-carbinol may be potential mechanisms.	Oral gavage daily for 4 weeks.	A cocktail containing neomycin (0.5 g/L), metronidazole (1 g/L), vancomycin (0.5 g/L), and ampicillin (1 g/L) for 7 days.	131
33	Aging-related disorders	C57BL/6 J mice (4 weeks)	C57BL/6 J mice (18 months)	FMT significantly ameliorates systemic diseases associated with natural aging, in particular playing a role in protecting the liver and improving glucose sensitivity, hepatosplenomegaly, inflammation, the antioxidant capacity, and intestinal barrier.	FMT increases the expression of tight junction proteins ZO-1 and occludin, and improves the function of the intestinal epithelial barrier. The general increases in the levels of *Akkermansia muciniphila*, acetic acid, and *A. mucophil* may be the underlying mechanism.	Oral gavage, qd for 1 week and qod for 11 weeks.	Antibiotic cocktail with 10 g/L metronidazole, 5 g/L vancomycin, and 10 g/L neomycin, while 1 g/L ampicillin was administered in the drinking water simultaneously for 5 days.	132
34	Skin aging	Male HR-1 hairless mice (2 months old)	GF mice (9 weeks old)	The skin moisture content of old mice is significantly lower than that of young mice, but the expression level of Aqp3 in the skin of old FMT mice is significantly higher than that of young FMT mice.	FMT does not decrease Aqp3 expression, but increases Aqp3 expression. Thus, the contribution of the gut bacteria to the downregulation of Aqp3 expression in older mice is low, taking other factors into account.	Oral gavage for 3 days.	GF mice	133
35		Male HR-1 hairless mice (15 months old)	GF mice (9 weeks old)					

Abbreviations: Aquaporin-3, Aqp3;Fecal microbiota transplant, FMT;Middle cerebral artery occlusion, MCAO;Short-chain fatty acids, SCFAs.

#### 3.4.3. Prospects and challenges for the clinical application of FMT

The clinical application of FMT has shown exciting results in the treatment of intestinal diseases,^100,102,134,135^ but the use of FMT for the treatment of osteoporosis is lacking. Its risks and ethical considerations may be the most important issues in promoting this burgeoning therapeutic regimen. Some reported adverse effects or potential risks and the corresponding solutions are described below.

In terms of risks, the most common adverse events were abdominal pain, diarrhea,^136–138^ nausea, dizziness, abdominal distension, aggravation of colitis, weight gain, vomiting, headache, weight loss, fever, anemia, constipation, and elevated alkaline phosphatase levels. The most common serious adverse events are *Clostridium difficile* infection,^136–138^ suicide,^137^ pneumonia,^134,138^ hepatic encephalopathy, sepsis, and intestinal perforation.^137^ In addition, feces give people an unpleasant experience in terms of vision and smell; therefore, the medical staff preparing the flora products and patients receiving treatment do not readily accept the application of feces to the human body from both psychological and physiological aspects. However, overall, no significant difference in adverse events was observed between FMT treatment and traditional treatment.

The possible causes of adverse reactions of FMT include the production method and administration route of enterobacterial products. Therefore, reasonable control of indications, strict screening of donors, standardization of the processing and production of intestinal bacteria products, and strengthening of quality control and supervision are practical means to reduce adverse effects. In addition, donor strain engraftment was enhanced through antibiotic pretreatment and bowel lavage.^139^ Moreover, colonoscopic administration, rather than oral administration, can also reduce gastrointestinal symptoms.^140,141^

With respect to ethical considerations, first, the management, attribution (jurisdiction right) and ethics of both FMT and fecal products are controversial because our understanding of the mechanism and therapeutic effect of FMT, an emerging therapeutic method, is limited. Therefore, in the application of decision-making and ethical considerations, researchers need to respect the principles of autonomy, be fully informed, be safe/do no harm, and ensure that the selection of donors and recipients is beneficial and fair.^142^ In practice, initiatives to protect both doctors and patients need to be implemented. For example, staff should wear face masks and eye masks; the preparation process should be performed in strict accordance with technical specifications; and oral administration, a nasogastric tube or colonoscopy should be individually selected for patients.

#### 3.4.4. Mini conclusions

Finally, although FMT has a wide range of medical therapeutic advantages and potential commercial applications, as an emerging treatment, guidance for standardized procedures for FMT and evaluations of the short- and long-term safety of this technology and related products are needed whether via clinical trials^143^ or animal studies.^144^

### 3.5. Other factors affecting the gut‒bone axis

As mentioned above, the GM is susceptible to both endogenous and exogenous influences. We briefly describe the effects of pharmaceuticals, diet, and exercise on the gut‒bone axis.

Drugs taken orally directly interact with the GM and interfere with its structure and composition. The role of the GM in drug metabolism has been widely studied and applied in clinical practice.^145–148^ However, studies of factors related to GM metabolism during osteoporosis therapy with drugs (e.g., bisphosphonates, PTH, calcitonin, and selective estrogen receptor modulators) are rare. Although no direct evidence of the effects of anti-osteoporosis drugs on the GM are available, a study that investigated changes in the oral microbiota after zoledronic acid application revealed that, at the genus level, the proportions of *Novosphingobium, Dubosiella, Mannheimia, Prevotella, Brevundimonas, and Bacteroides* increased after zoledronic acid administration.^149^ Another important study showed that parathyroid-dependent bone formation requires butyrate production by the GM.^150^ The heterogeneity of the therapeutic effects of current anti-osteoporosis drugs^151–153^ suggests that drug metabolism requires the GM. Therefore, further study of the role of the GM in anti-osteoporosis drug metabolism may be an important way to overcome bottlenecks in drug therapy.

Diet is an intervention factor that directly affects the GM and has been widely studied for its effect on bone health. High-fat diets lead not only to obesity but also to bone loss. In addition to the administration of probiotics, prebiotics, and dietary supplements, bone loss can be reduced by reversing GM dysbiosis.^154–156^ In addition to the use of probiotics, a balanced diet (including minerals, dairy products, fruits and vegetables), optimal protein and calcium intake, and adequate vitamin D intake are important for maintaining bone health and preventing fragility fractures.^157–160^

Exercise is one strategy for preventing osteoporosis. High-intensity interval training increases the alpha diversity and *Bacteroidetes/Firmicutes* ratio of the distal intestinal and fecal microbiota.^161^ Exercise also affects the physiological functions and metabolic activities of various parenteral organs, and its influence is comprehensive. In addition, different exercise methods, frequencies and intensities influence the intestinal flora in numerous ways.^162–164^ Some reviews have summarized the moderating effects of exercise on the GM and osteoporosis.^164,165^ One interesting study examined changes in the GM and skeletal system using both a high-fat diet and exercise as variables. Additionally, one study showed that exercise can reverse the structural changes in the GM and bone loss caused by a high-fat diet.^166^ This study investigated the effects of diet and exercise interventions on the GM and bone mass, respectively, and revealed the dynamic changes in the GM and its susceptibility to external influences.

In summary, healthy diets and exercise represent lifestyle strategies to prevent osteoporosis. The effect on the GM may be just one mechanism by which these interventions achieve this outcome. Importantly, diet, nutrient absorption, and the effects of exercise on organs outside the GI tract should be considered.

## 4. Metabolism and the gut microbiota

### 4.1. Bile acids and bile acid receptors

BAs are important components of enterohepatic circulation^167^ that are produced in the liver and are the main component of mammalian bile.^168^ BAs can be divided into primary BAs (cholic acid (CA), glycodeoxycholic acid (GDCA), taurocholic acid (TCA), and glycocholic acid (GCA)) and secondary BAs (deoxycholic acid (DCA), lithocholic acid (LCA), etc.). Currently, BAs are recognized as a new type of signaling molecule that acts by binding to specific receptors, among which secondary BAs are more abundant, more extensive in function, and receive more attention.^169,170^

A single-center cross-sectional study of 150 postmenopausal women revealed that serum total bile acid (TBA) levels were positively associated with the BMD of the lumbar spine, femoral neck, and total hip and negatively associated with bone turnover biomarkers that reflect bone resorption.^171^ Another single-center retrospective study involving 2,490 Chinese adults aged 20–59 years also confirmed that serum TBA levels were positively associated with femoral neck BMD.^172^ However, in a special group of patients with type 2 diabetes mellitus (T2DM), TBA and BMD are negatively correlated.^173^ The possible reason is that the pathway by which bile acid regulates bone metabolism may be complicated by the pathophysiological mechanism of diabetes. One study investigated differences in the levels of individual BAs in women with high/low BMDs (65 vs. 71 patients aged 20–40 years) and reported that ursodeoxycholic acid (UDCA) and tauroursodeoxycholic acid (TUDCA) were correlated with a low BMD (OR = 2.69 (1.48–5.41), *p* = 0.0025; OR = 2.18 (1.3–3.88), *p* = 0.0049).^174^ These findings suggest a potential link between BA metabolism and bone metabolism.

Hepatic bone disease is a special type of osteoporosis that manifests as a decrease in osteogenic ability.^175^ The addition of bilirubin and LCA decreased the expression of BMPs, RUNX2 and antiapoptotic genes and upregulated proapoptotic genes in human osteosarcoma cells *in vitro* .^176^ The results obtained from bone cells (MLO-Y4 and MLO-A5 cell lines) and human bone fragments also support this conclusion.^177^ Other experiments have shown that bilirubin promotes the differentiation of RAW 264.7 cells and human peripheral blood mononuclear cells (PBMCs) into osteoclasts and reduces osteoclast apoptosis, but LCA has the opposite effect.^178^

UDCA counteracts the damaging effects of bilirubin or LCA on osteoblast viability, proliferation, and mineralization.^179,180^ Another secondary bile acid, TUDCA, has also been reported to reduce bone loss in OVX mice.

Currently, the identification of new bile acids,^181^ as well as the continuous updating of knowledge about bile acid modifications,^182^ provides important guidance for future research on the effects of bile acids on bone metabolism.

**Figure 1.**
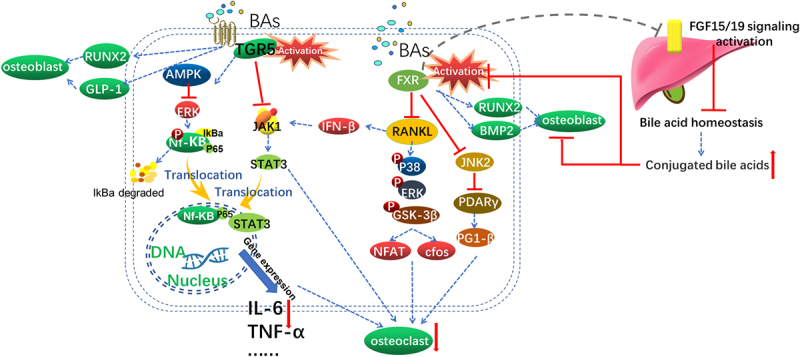
Bile acids activate the bile acid receptors TGR5 and FXR to regulate bone metabolism.

Bile acid receptors are divided into two categories: nuclear receptors, including farnesol X receptor (FXR), vitamin D receptor (VDR), pregnane X receptor (PXR) and constituent androstane receptor (CAR), and membrane receptors, which mainly include the Takeda G protein-coupled receptor 5 (TGR5) and the 1-phospho-sphingosine receptor 2 (S1PR2).^183,184^ These receptors are widely expressed in all body tissues and form the core of bile acid signaling. The effects of FXR and TGR5 on bone metabolism and the underlying mechanism are described below (Figure 1).

TGR5 activation promotes osteoblast production by upregulating RUNX2 and GLP1 expression. TGR5 activation can also reduce osteoclast generation by inhibiting the expression of NFKB- and JAK-STAT3 pathway-related inflammatory factors. FXR activation promotes osteoblast generation by upregulating the expression of RUNX2 and BMP2. When bile acid metabolism in the liver is disturbed, the levels of chelated bile acids increase and they act as natural inhibitors of FXR, thereby inhibiting osteogenic function. FXR activation also decreases osteoclast generation by inhibiting the P38 and JNK pathways.

#### 4.1.1. FXR

Farnesol X receptor (FXR) is expressed in bone, especially in osteoblasts.^185^ Compared with wild-type mice (FXR^+/+^), male mice deficient in FXR (FXR^−/−^) present a significant increase in osteoclast number and osteoclast surface area and a significant decrease in BMD. However, the BMD of female FXR^−/−^ mice is not significantly different from that of female FXR^+/+^ mice. After oophorectomy, bone loss is accelerated in female FXR^−/−^ mice compared to that in ovariectomized FXR^+/+^ mice.^186^

Initially, researchers found that FXR activation stimulates RUNX2-mediated BMSC osteoblast differentiation, whereas its inhibition leads to an adipocyte-like phenotype.^187^ Furthermore, the use of fexaramine, a selective receptor agonist of FXR, inhibits RANKL-induced osteoclast formation and reduces bone resorption both *in vivo* and *in vitro*. The possible mechanism involves blocking the phosphorylation of p38, ERK and GSK3β triggered by RANKL, resulting in the inhibition of NFATc1 and c-fos expression. Interestingly, the authors used BMMs from FXR^−/−^ mice and observed that the absence of FXR does not affect the anti-osteoclastic effect of fexaramine. Thus, the effect of fexaramine on RANKL-induced osteoclast formation is independent of FXR.^188^ However, another *in vitro* study revealed that the use of FXR receptor agonists enhances the differentiation of BMP-2-induced MSCs (mouse bone marrow-derived mesenchymal stem cell-like ST2 cells, ST-2 MSCs) into osteoblasts by activating FXR and inducing the expression of RUNX2.^189^

In addition, the use of geniposidic acid (GPA), a natural agonist of FXR, has been shown to induce RUNX2 expression and improve osteogenic function both *in vivo* and *in vitro*.^185^ This study also revealed that the ability of GPA to enhance osteogenesis occurs in an FXR-dependent manner and that the absence of FXR leads to the disappearance of the bone-promoting effect of GPA.

The FXR/FGF15 axis, the gut – liver – endocrine axis, may participate in ABX-induced GM dysbiosis and bone loss. Specifically, after minocycline administration, the FXR/FGF15 axis and bile acid homeostasis are disrupted, and the levels of conjugated BAs are subsequently increased. These bile acids are known as FXR antagonists; as a result, FXR signaling is weakened, osteoblast function is inhibited, and bone mass, bone microstructure and fracture resistance are impaired.^190^ The BA-FXR-FGF15/19 axis is involved in mediating host skeletal muscle loss.^191^

FXR also affects bone mass by regulating osteoclastic activity. In an osteoarthritis mouse model, FXR receptor agonists attenuate osteoclast fusion in subchondral bone by inhibiting the JNK1/2/NFATc1 pathway.^192^ Another study using offload and OVX mouse models showed that FXR deficiency accelerates osteoclast formation by downregulating JNK1/2 expression and increasing the expression of peroxisome proliferator-activated receptor (PPARγ) and peroxisome proliferator-activated receptor γ coactivator 1 (PGC-1β). In addition, FXR deficiency downregulates the expression of interferon-β (IFN-β) through receptor activator of nuclear factor-kappa B ligand (RANKL), thereby impacting the downstream JAK3-STAT1 signaling pathway. This process in turn increases osteoclast formation.^193^

In tumor-induced bone resorption, the use of FXR antagonists inhibits RANKL- and tumor cell (human breast tumor cells [MDA-MB-468] or human multiple myeloma [U266]) induced osteoclast generation by inhibiting NF-κB activation.^194^ FXR is also expressed in MCF-7 (estrogen receptor (ER)-positive) cell lines of breast cancer origin. FXR activation mediates ER activation and promotes cell proliferation, but this effect is lost when estrogen is inhibited.^195^ In a follow-up analysis of 81 patients with distant breast metastases, this team confirmed a strong correlation between FXR expression in primary breast tumors and the development of bone metastases.^196^ The results of this series of studies suggest that the use of FXR agonists in cancer patients may promote tumor growth and bone metastasis.

#### 4.1.2. TGR5

Takeda G protein-coupled receptor 5 (TGR5)-deficient (TGR5^−/−^) female mice have a bone mass close to that of wild-type (TGR5^+/+^) female mice in young adulthood and middle age but exhibit increased levels of osteoclast differentiation, which leads to accelerated bone loss in old age (7 months) or after ovariectomy.^197^

A series of studies of TGR5 indicated that activating TGR5 can reduce inflammation.^198–200^ Evidence from an animal study in OVX mice confirmed that TGR5 is associated with the expression of systemic inflammatory factors and is involved in bone metabolism.^72^ Additionally, TGR5 activation increases bone mass by downregulating the JAK1-STAT3 signaling pathway to reduce the levels of IL-6 and TNF-α.^201^ Folic acid supplementation increases the level of LCA and the expression of the TGR5 gene, increases the phosphorylation of AMPK, and decreases the phosphorylation of NF-κB and ERK, thereby reducing bone loss.^202^

TGR5 does not appear to have a significant effect on osteogenic function *in vivo* ,^197^ but *in vitro* experiments have shown the opposite results; namely, TGR5 activation induces RUNX-2 expression, osteoblast differentiation and mineralization by activating the AMPK pathway.^203^

#### 4.1.3. S1PR2

The S1PR2 receptor was originally found to be involved in hepatic lipid metabolism^204^ and liver disease.^205–208^ Conjugated bile acids activate the ERK1/2 and AKT signaling pathways primarily through S1PR2.^204,209^ In addition to bile acids, another important ligand of S1PR2 is sphingosine-1-phosphate (S1P). S1PR2 is also expressed in osteoblasts and osteoclasts.^210^ In response to the administration of S1P, S1PR2 coordinates with S1PR1 to regulate the dynamic migration of osteoclast progenitor cells (OCPs), where S1PR1 directs positive chemotaxis toward S1P and S1PR2 mediates negative chemotaxis.^211^ Therefore, mice with a S1PR1 gene deletion present osteoporosis,^212^ but mice with a S1PR2 gene deletion present moderate osteopetrosis.^211^

As an osteoclast – osteoblast coupling factor, S1P is both an intracellular messenger and an extracellular signaling molecule.^213,214^ Osteoclast-derived S1P has been shown to stimulate mesenchymal stem cell movement *in vitro* by activating the FAK/PI3K/AKT signaling pathway via S1PR2.^215,216^ S1P also affects osteoblast differentiation and activity in an autocrine manner. S1PR2 antagonists decrease osteoblast differentiation and inhibit osteoclast generation by decreasing the OPG/RANKL ratio, whereas S1PR2 agonists increase the osteoblast number and activity in mice.^217,218^ Notably, the current mainstream view is that the regulatory effect of S1PR1/2 on bone metabolism mainly depends on S1P. Therefore, studying the effect of the bile acid – S1PR1/2 pathway on bone metabolism may be a future research direction.

#### 4.1.4. VDR, PXR and CAR

Pregnane X receptor (PXR), constitutive androstane receptor (CAR) and vitamin D receptor (VDR) are closely related nuclear receptors that play crucial roles in BA homeostasis.^219–221^ Moreover, these nuclear receptors, including FXR, are involved in the expression of genes that directly regulate and encode key proteins involved in drug and bile acid transport.^222^

In bone metabolism, CAR was first shown to be associated with BMD in postmenopausal women and rat primary osteoblasts and is believed to be involved in the progression of osteoporosis.^223^ Further studies revealed that CAR-deficient mice present increased bone mass, possibly due to reduced testosterone metabolism due to Cyp2b downregulation rather than reduced osteogenic differentiation or increased osteoclast numbers.^224^ In addition, glycolithocholic acid (GLCA), a metabolite of the intestinal microbiota, enhances the differentiation of CD4 T cells into Tregs by activating CAR. The increased frequency of Tregs promotes the osteogenic differentiation of bone marrow mesenchymal stem cells, thereby alleviating osteoporosis.^225^

PXR can be activated by exogenous substances or drugs and is involved in bone metabolism by regulating CYP24 gene expression and altering vitamin D (3) hormone activity and calcium homeostasis.^226^ PXR knockout mice present a decreased bone mass, reduced bone formation, enhanced bone resorption,^227^ and aging-dependent wearing of articular cartilage.^228^ A recent study revealed that *C. sporogenes* and its derived metabolite indole-propionic acid (IPA) can. By inhibiting the ubiquitination and subsequent degradation of pregnane X receptor (PXR) induced by RANKL, osteoclast differentiation and function are inhibited.^34^

VDR is a widely expressed nuclear receptor with multiple physiological effects. As the most well-known bone metabolic pathway, the 1,25(OH) _2_D_3_/VDR signaling pathway not only plays a role in the maintenance of mineral homeostasis^229^ and iliac mineralization^230^ but also directly acts on osteoblasts, chondrocytes and osteoclasts through paracrine or autocrine signaling to play a direct role in bone metabolism.^231,232^ In addition to 1,25(OH) _2_D_3_ and its analogues, VDR, a bile acid-activating receptor, can be activated by the secondary bile acid LCA^233^ and promote osteoblast differentiation.^234^ However, LCA accumulation has devastating effects on osteosarcoma cells^176^ and osteocytes.^177^ Moreover, LCA also downregulates the effects of vitamin D on osteoblasts.^235^ However, systematic studies on the bile acid – VDR – bone metabolism axis are lacking.

##### Mini conclusions

In conclusion, BAs and their receptors play important regulatory roles in bone metabolism, possibly through the regulation of inflammatory factors and osteoclast activation. Interestingly, the absence of both FXR and TGR5 may amplify bone loss under pathological conditions (post-OVX). These findings suggest that pathological changes in some important systems, functions, and microenvironments may trigger bone loss through bile acid receptors and their pathways. In the future, exploring the metabolomic changes in individuals with specific bile acid receptor deletions under pathological conditions, especially changes in the bile acid spectrum, may be one approach to address this question. Another potential research direction involving the development of drugs targeting both TGR5 and FXR is a promising strategy for osteoporosis treatment, as described in previous studies.^197^

### 4.2. SCFAs

SCFAs are saturated fatty acids with fewer than six carbon groups attached to carboxylic acids. In the human body, the three most common SCFAs are acetate (C2), propionate (C3), and butyrate (C4). SCFAs can be produced by beneficial bacteria in the gut through the digestion and fermentation of dietary fiber. C2 can be produced by many types of bacteria, but C3 and C4 can be produced by only certain types of bacteria. For example, C4 is derived mainly from *Prausnitzii Faecalibacterium*, *Eubacterium*, *Halebacterium* and *Bromococcus* .^236–238^ The content and composition of SCFAs are affected by the type of plant fiber ingested, the residence time of plant fiber in the intestine, and the composition of the GM. SCFAs are also important energy sources and regulatory molecules for microorganisms and humans. Among them, acetic acid reaches the liver through the portal vein and is absorbed for gluconeogenesis and fat synthesis. Propionate and butyrate can provide energy for colon cells,^239^ and propionate can participate in the synthesis of glucose by liver cells.^240,241^

**Figure 2.**
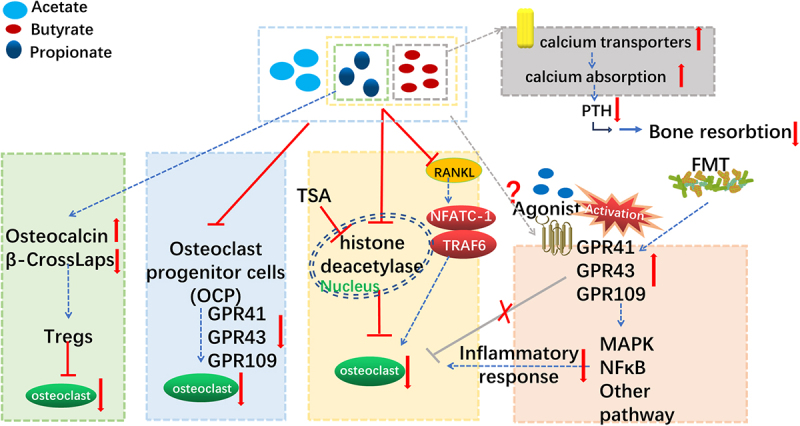
Potential mechanisms by which SCFAs and their receptors affect bone metabolism.

SCFAs may play a role in bone metabolism (Figure 2). A meta-analysis revealed that, compared with those in healthy people, the abundances of SCFA producers, including members of the genera *Collinsella, Megasphaera, Agathobaculum, Mediterraneibacter, Clostridium XIV*, and *Dorea*, are significantly reduced in osteoporosis patients.^242^ According to a study comparing old and young mice, *Akkermansia* and *Parabacteroides* are abundant in the GM of young mice but are less abundant in older mice. Three pathways (lysine degradation, fatty acid biosynthesis, and butyrate metabolism) are involved in the biosynthesis of SCFAs, such as butyrate, which is decreased in aged mice.^26^ In addition, in a cross-sectional study of 236 healthy children, pelvic BMD and total body (less than the head) BMD were significantly positively associated with the levels of acetic acid, butyric acid, valeric acid, hexanoic acid, and total SCFAs.^243^

Propionate upregulates osteocalcin, downregulates β-crossLaps, and downregulates Tregs. A mixture of SCFAs inhibits the conversion of OCPs into osteoclasts. A mixture of propionic acid and butyrate inhibits osteoclast maturation by inhibiting histone deacetylases and RANKL. SCFAs also regulate calcium homeostasis and the expression of inflammatory factors to affect bone metabolism.

C3 and C4 inhibit osteoclast differentiation by inhibiting histone deacetylases (HDACs).^244,245^ The HDAC inhibitor TSA significantly inhibits osteoclast differentiation, but the agonists GPR41, GPR43 and GPR109A (the receptors of SCFAs) have no effect. 3-Hydroxybutyrate acid (3HB) downregulates nuclear factor of activated T-cell cytoplasmic 1 (NFATc1), which is a transcription factor involved in preosteoclast differentiation.^246^ *In vitro* experiments have shown that 3HB can promote calcium deposition in MC3T3-E1 cell lines in a dose-dependent manner, and *in vivo* experiments have also shown that 3HB can reduce bone loss in OVX mice, increase the maximum load of the femur and bone deformation resistance, and increase the trabecular bone volume.^247^

In addition, intragastric administration of SCFAs (a mixture of 67 μM acetate, 26 μM propionate, and 40 μM butyric acid) significantly improves the bone volume/tissue volume ratio, bone thickness, and bone mineral density in OA model mice by directly affecting bone marrow-derived osteoclast progenitor (OCP) cells.^248^

SCFAs affect osteoclast differentiation by regulating energy metabolism. Differentiation from precursors to mature osteoclasts depends on oxidative phosphorylation, whereas bone resorption in mature osteoclasts depends on glycolysis. However, *in vitro* experiments have shown that, after C3 and C4 treatment, the metabolism of osteoclast precursors shifts to glycolysis at an early time point during osteoclast differentiation, thus affecting osteoclast differentiation.^249^

SCFAs represent an important way by which FMT affects bone mass. After the transfer of *Prevotella* spp. to young mice via FMT, recipient mice presented decreased SCFA levels, increased numbers of osteoclasts, and a reduction in total bone mass.^249^ In another experiment involving FMT treatment, transplanting feces from healthy mice into OVX mice optimized the composition and abundance of the GM, increasing fecal SCFA (mainly acetic acid and propionic acid) levels.^110^ Similarly, another FMT study demonstrated that transplanting the GM from healthy mice restored the levels of the damaged bacterial metabolites SCFA (butyrate, valerate, and propionate) and reduced bone loss.^250^ These studies confirmed that FMT may be a potential approach for restoring SCFA levels and treating osteoporosis.

SCFAs and their receptors act as bone resorption modulators.^251^ Endogenous receptors of SCFAs, including GPR18, GPR41, GPR43 and GPR109A, are expressed in both osteoclasts and osteoblasts.^252,253^ Compared with wild-type controls, GPR109A-deficient (GPR109A^−/−^) mice present significantly greater bone mass and strength in the tibia and spine, as well as significantly lower osteoclast counts and bone absorption marker levels.^254^ Even in gonadectomy (OVX) and orchidectomy (ORX) osteoporosis models, the degree of bone loss in GPR109A^−/−^ mice is lower than that in wild-type mice, and the expression of a bone resorption marker (Cathepsin K) is significantly lower in GPR109A^−/−^ mice than in wild-type mice.^255^ After FMT, GPR41 and GPR43 mRNA levels are significantly increased in the tibia, as was the bone mass.^250^ The inhibitory effects of C2/C3/C4 on osteoclast differentiation and bone resorption are independent of the free fatty acid surface receptors FFA3 (GPR41) and FFA2 (GPR43).^249^ The possible mechanism for this effect may be that C3 and C4 significantly inhibit TRAF6 and NFATc1 at early time points after RANKL receptor activation. The upregulation of the SCFA receptors GPR43, GPR41 and GPR109A in osteoclast progenitor cells may lead to increased downstream signal transduction. When OCPs differentiate into osteoclasts, the expression of GPR43 and GPR41 is significantly downregulated, whereas the expression of the butyrate-specific receptor GPR109A is downregulated.^248^

In addition to its effects on osteoporosis, butyrate has been shown to inhibit osteoclast activity *in vitro*, regulate systemic inflammation in mice, and promote fracture healing *in vivo*.^256,257^

Among patients with abnormal bone metabolism after laparoscopic sleeve gastrectomy, those with greater changes in the intestinal microbial composition after surgery have greater bone loss. The possible mechanism underlying this outcome is that gut microbes reduce butyrate production, which in turn leads to lower IGF-1 levels.^258^

In addition to modulating osteoclast activation, supplementation with propionic acid has been shown to increase osteocalcin levels and decrease β-CrossLaps levels. A significant positive correlation between the level of osteocalcin and the number of peripheral regulatory T cells (Tregs) has been observed.^259^ Another study reported that the number of Tregs increased after SCFA treatment,^249^ and Tregs were shown to inhibit osteoclasts and increase bone mass.^260,261^ Therefore, an increase in the number of Tregs may be one of the mechanisms underlying the bone-protective effect of SCFAs and may explain the improvements in bone mass.

Insulin-like growth factor 1 (IGF-1) is a hormone known to play a role in bone growth. In GF mice that received FMT, the serum levels of IGF-1 increase significantly, which promotes bone formation.^91^ Another FMT study also revealed increases in bone mass and the level of IGF1 ^110,231^. These results suggest that SCFAs may indirectly participate in regulating bone mass by influencing IGF-1.

SCFAs can also interact with GPR43 to inhibit the expression of LPS-induced cytokines such as TNF-α and interferon gamma (IFN-γ), increase the expression of IL-4 and IL-10, and induce the activation of Treg cells in the colon, reducing the production of inflammatory cytokines and relieving intestinal inflammation.^262–264^ SCFAs improve the solubility of minerals in the gut and promote calcium absorption by lowering the pH of the gut. Butyrate increases the expression of intracellular calcium transporters.^265^ The expression of these transporters drives an increase in intracellular calcium absorption, which can limit the production of parathyroid hormone and substantially reduce bone resorption.^266^

#### Mini conclusions.

The type and content of SCFAs are associated with changes in the GM during osteoporosis. SCFAs and their endogenous receptors not only directly act on osteoblasts and osteoclasts but also participate in the regulation of bone metabolism by affecting host immunity, metabolism and mineral absorption. In addition, SCFAs are also involved in the role of FMT in regulating bone mass, and additional supplementation with SCFAs has similar effects.

### 4.3. Trimethylamine N-oxide (TMAO)

Trimethylamine (TMA) is derived from foods (especially red meat, milk, and eggs). Large amounts of TMA are absorbed by the gut, enter the bloodstream, and then enter the liver. Small amine compounds are produced by oxidation mediated by the liver-flavin monooxygenase (FMO) family.

TMAO is often associated with cardiovascular disease and chronic kidney disease. TMAO promotes vascular calcification and renal fibrosis through a variety of complex mechanisms, such as inflammation, oxidative stress and apoptosis, and the level of TMAO in blood is positively correlated with cardiovascular events.^267–269^ Moreover, studies^270–272^ have shown that chronic kidney disease is accompanied by significant changes in the GM, resulting in increased production of bacteria-derived uremic toxins (such as indolol sulfate, para-cresol sulfate and TMAO), whereas breakdown of the intestinal epithelial barrier also accelerates the transfer of TMAO into the systemic circulation, resulting in adverse effects.

TMAO also damages bone health. Clinical studies have shown that the blood TMAO concentration is significantly negatively correlated with BMD. *In vitro*, TMAO promotes the lipogenic differentiation of BMSCs, inhibits osteogenic differentiation, and increases proinflammatory cytokine levels through the NFκB pathway.^273^ Another animal study confirmed that after OVX, the structure of the GM changed, accompanied by an increased TMAO content. The use of gold nanospheres significantly altered the diversity and composition of the gut microbiota, specifically reducing the abundance of TMAO-associated strains and ultimately reversing bone loss after ovariectomy.^274^

### 4.4. Microbial – host isoenzymes (MHIs)

As mentioned above, most of the current research on the mechanism of action of gut microbes has focused on their associated small-molecule metabolites, but other large molecules, such as proteins, have not been studied. The concept of MHIs has recently been proposed, and their role in mediating microbiome – host interactions has attracted much attention. MHIs are enzymes produced by the GM that can perform similar functions in the host. MHIs link microbial enzyme activity to host physiological functions.

For example, bacteria-derived DPP4 isoenzymes can enter intestinal tissue under conditions of intestinal barrier damage and affect glucose tolerance by degrading GLP-1. Host DPP4 inhibitors may not be effective at targeting bacterial DPP4. This finding explains the heterogeneous responses to sitagliptin in clinical treatment. Based on this information, the team selected Dau-4 as a drug that selectively inhibits bacterial-derived DPP4 while not interfering with host DPP4 activity and achieved excellent therapeutic effects.^146^ Researchers have identified an interspecies pathway of the GM and bacterial-derived enzymes involved in the metabolism of L-DOPA, namely, the conversion of L-DOPA to dopamine via pyridoxal phosphate-dependent tyrosine decarboxylase from *Enterococcus faecalis*, followed by the conversion of dopamine to m-tyramine via molybdenum-dependent dehydroxylase from *Eggerthella lenta* .^275^ The results also suggest that these changes in microbial activity may contribute to the heterogeneous response of patients to the drug levodopa. Another study of castration-resistant prostate cancer (CRPC) suggested that *Ruminococcus* spp. *DSM_100440* and/or *OM05_10BH* were more abundant in patients with higher serum testosterone levels. Using ABX to deplete the gut microbiota can reverse this phenomenon.^276^ This study suggested that the GM could be an alternative source of androgens and contribute to the potential risk of prostate cancer growth or endocrine resistance.

These findings suggest that gut microbes regulate physiological functions by producing specific enzymes. Precisely targeting natural host enzymes and intestinal microbial isoenzymes to treat or intervene in metabolic diseases is a personalized treatment method.

In fact, MHI is widespread in the gut^275,277^ and has a variety of nonmetabolite-dependent functions, but its role in host metabolic diseases is unclear. As another bridge between the microbiome and its host, MHIs deserve further investigation. Mechanistic research and precision treatment of bacteria-derived enzymes based on interdisciplinary cooperation and advanced sequencing technology are future areas of research to explore.

## 5. Other mechanisms

The GM also regulates bone mass by regulating the absorption of nutrients, such as calcium, phosphorus, and vitamin D. In addition, changes in the composition of the GM can affect intestinal permeability and intestinal barrier function, and intestinal mucosal barrier dysfunction may lead to elevated serum LPS levels, resulting in bone loss. Gut microbes are capable of regulating the levels of host hormones, such as neurotransmitters,^278^ estrogen,^279^ testosterone,^280,281^ thyroid hormones,^282^ and growth hormone,^283^ which have been shown to be involved in the regulation of bone metabolism. Moreover, damage to the gut barrier may not be caused primarily by changes in the composition of the gut microbiota. Changes in intestinal NO homeostasis, especially increases in arginase activity and decreases in NO bioavailability in the intestinal mucosa, may lead to the loss of intestinal compact linking proteins.^123^

Bone metabolism – microbes – immunology is another emerging interdisciplinary discipline based on bone immunology, as these three important systems share many regulatory molecules, forming complex, deep synergistic networks that work together to maintain homeostasis in the internal environment. The GM affects bone mass by triggering an immune response in the gut locally or throughout the body, uniting immune cells, regulating proinflammatory/anti-inflammatory cytokines, and promoting cytokine production by osteoclasts/osteoblasts.

## 6. Conclusions and prospectives

Current research dilemmas and potential directions are focused on a deeper sequencing depth and functional exploration. Specifically, First of all, current research dilemma and potential directions are focused on deeper sequencing depth and functional exploration. To be specific, for example, high-throughput sequencing equipment/technology and algorithm/AI are needed to support the discovery of new strains,^284,285^ genome and functions of GM.^286–288^ Besides, novel metabolites of GM are also worth exploring, for example, exploring new bile acids with click chemistry techniques.^72^ Second, the effect of specific strain or metabolite in the host’s pathophysiological process, as well as the impact of systemic metabolism, also need to be fully explored. For example, the function and mechanism of probiotics could be found through whole genome sequencing technology, in vitro experiments, in vivo experiments, animal experiments and clinical trials. It is also worth comparing the advantages and efficacy of monostrain and multistrain probiotics.^289^ Third, FMT is an important research method, which is worthy of in-depth study. The potential research direction including 1). the type selection, dosage optimization and transplantation methods selection of transplanted bacteria; 2). evaluation of colonization status and adaptability of transplanted bacteria; 3). optimal conditions of the intestinal environment (including pH value of intestinal microecology, oxygen concentration, bile acid concentration, and intestinal peristalsis); 4). optimal conditions of host (including immune status, intestinal barrier function, and preexisting intestinal flora composition of host); 5). evaluation of the effect of other conditions on transplant efficiency (including diet, genetics, medical intervention, etc.). Forth, the improvement of safety and effect of FMT and the establishment of evaluation standards, as well as the process optimization and standardization of intestinal bacteria products and industrial management. Last but not least, the discovery of microbial – host isoenzymes^146^ adds a new perspective to the dialog between bacteria and human body, which is of great significance in the study of the heterogeneity of drug metabolism.

In summary, currently, correlations between the GM and bone metabolism are receiving increasing attention. Studies on the mechanisms underlying the relationship between the GM and bone metabolism are interdisciplinary and promising and are the focus of current and future research. Based on current knowledge, restoring the balance of the gut microbiota, transplanting a healthy gut microbiota or supplementing a healthy gut microbiota with a specific species are predicted to improve bone health. However, the mechanism of the GM and its potential risks and ethical considerations remain primary questions that need to be fully explored before the gut microbiota and related products can be applied in clinical settings. In addition, the gut microecology is dynamic and complex and is easily affected by both individual and external factors. When studying the intestinal microbiota, especially in human studies under complex realistic environments, researchers must be cautious and thoughtful about the results. Further study of the gut microbiota and its influencing factors and mechanisms in bone health and deeper knowledge of the gut – bone axis are highly important for targeting bone health with a healthy gut microbiota.

## Abbreviations

3HB: 3-Hydroxybutyrate acid
ABT: Antibiotic treatment
ABX: Antibiotic cocktail
Bas: Bile acids
BDNF: Brain-derived neurotrophic factor
BMD: Bone mineral density
CA: Cholic acid
CAR: Constituent androstane receptor
CRPC: Castration-resistant prostate cancer
DCA: Deoxycholic acid
DIO: Disuse-induced osteoporosis
DXA: Dual-energy X-ray absorptiometry
ER: Estrogen receptor
FMO: Flavin monooxygenase
FMT: Fecal microbiota transplantation
FXR: Farnesol X receptor
GCA: Glycocholic acid
GDCA: Glycodeoxycholic acid
GF: Germ-free
GIOP: Glucocorticoid-induced osteoporosis
GLCA: Glycolithocholic acid
GM: Gut microbiota
GPA: Geniposidic acid
HDCA: Histone deacetylase
IFN-β: Interferon-β
IFN-γ: Interferon gamma
IGF-1: Insulin-like growth factor 1
IPA: Indole-propionic acid
LCA: Lithocholic acid
LPS: Lipopolysaccharides
MHIs: Microbial – host isoenzymes
NFATC1: Nuclear factor of activated T-cell cytoplasmic 1
NPAM: Nonprematurely aged mice
NPY: neuropeptide Y
OCPs: Osteoclast progenitor cells
ORX: Orchidectomy
OVX: Ovariectomized
PAM: Prematurely aged mice
PBMCs: Peripheral blood mononuclear cells
PGC-1: Peroxisome proliferator-activated receptor γ coactivator 1
PPARγ: Peroxisome proliferator-activated receptor γ
PXR: Pregnane X receptor
RANKL: Receptor activator of nuclear factor-kappa B ligand
RCT: Randomized controlled trial
S1P: Sphingosine-1-phosphate
S1PR2: 1-Phospho-sphingosine receptor 2
SCFAs: Short-chain fatty acids
T2DM: Type 2 diabetes mellitus
TBAs: Total bile acids
TCA: Taurocholic acid
TGR5: Takeda G protein-coupled receptor 5
TMA: Trimethylamine
TMAO: Trimethylamine N-oxide
TUDCA: Tauroursodeoxycholic acid
UDCA: Ursodeoxycholic acid
VDR: Vitamin D receptor
